# Focal Seizures in a Child Receiving Terbutaline Sulfate: A Case Report

**DOI:** 10.7759/cureus.43540

**Published:** 2023-08-15

**Authors:** Falah Nadeem, Maria Ali, Nida Hameed

**Affiliations:** 1 Pediatrics, Rashid Latif Medical College, Lahore, PAK; 2 Pediatrics, Services Hospital, Lahore, PAK; 3 Obstetrics and Gynecology, Services Hospital, Lahore, PAK

**Keywords:** adverse effect, children, side effects, focal seizures, seizures, terbutaline

## Abstract

Terbutaline sulfate is a beta-adrenergic receptor agonist. More specific for B2 receptors, it is used as a bronchodilator in asthma. Its known side effects can include dizziness, tremors, and tachycardia. However, seizures are not among the commonly reported side effects. This is the case of a five-month-old girl who presented with focal seizures after the intake of terbutaline sulfate syrup. Other causes of the seizures were excluded through history and investigations, including an EEG and electrolyte panel. The seizures stopped on cessation of the terbutaline sulfate with no recurrence, leading us to believe that the focal seizures were an adverse effect of the terbutaline sulfate. A high index of suspicion for drug-related adverse effects should therefore be kept for a child with new onset focal seizures.

## Introduction

Beta receptors comprise a part of the body’s sympathetic system. Present on smooth muscle cells in the airways, B2 receptors play a major role in the management of asthma and chronic obstructive pulmonary disease [1]. Terbutaline is among the short-acting B agonists, with a duration of action of seven hours [2]. Its reduced action on B1 receptors makes its cardiovascular side effects minimum [2]. However, it is not recommended in children under the age of 12 years [3], although historically it has been shown to be effective in younger age groups without major side effects or tachycardia in single doses of 0.075 mg/kg [4]. It is available in powdered formulations for inhalation and injectable or subcutaneous and oral formulations for the management of asthma and bronchospasm [3].

## Case presentation

A five-month-old girl weighing 4.5 kg presented to the clinic with the complaint of shaking in the lower legs for three days. She was born prematurely at 33 weeks due to preeclampsia in the mother at a birthweight of about 1.6 kg and Apgar scores of 7 and 9. She was kept in an incubator for five days under observation due to her low birth weight but had no complications during that time. History was significant for a chest infection at the age of four months and two weeks, with one case of febrile seizures at a fever of 102 °F. After the typical generalized tonic-clonic febrile seizure, lasting about 30 seconds, the temperature was brought under control and antibiotics administered. There were no further episodes. Family history was positive for febrile seizures in several cousins.

The new onset shaking began four days ago and was limited to the lower limbs. It had increased in frequency from once a day to several times a day over the past 24 hours. A video recording of the activity was brought for inspection. A focal seizure in the lower limb was observed, which lasted for a few seconds, and vitals, including temperature, were found to be normal with the exception of a heart rate of 162 bpm. Blood sugar was 75 mg/dl, and the seizures were self-limited. The seizures occurred in the child's sleep and during wakefulness, and they were not stopped by holding the foot.

**Video 1:** Focal seizure The seizure is shown to be occurring in the lower limb, in self-limited, short episodes. No effect of grabbing the foot.

On further examination, no significant findings were discovered. The patient was found to be taking a single medication, a syrup containing terbutaline sulfate 0.3 mg/ml three times a day, for a lingering cough. She was being administered 1 ml for each dose, which had been raised to 2 ml by the mother in an attempt to have a better effect. The syrup was discontinued, and the patient referred to a tertiary care hospital for further investigation to rule out the causes of focal seizures. Investigations, including an electrolyte panel, complete blood count (CBC), and EEG, were performed. On the reports, the WBC count was 6,750/mm³, and other than iron deficiency anemia (mean corpuscular volume (MCV) 60.9 fL and serum ferritin 7.01 ng/ml), no abnormalities were discovered. There was no recurrence of seizures after discontinuing the terbutaline sulfate.

## Discussion

There have been only two previously reported cases of focal seizures caused by terbutaline [5,6] along with a single reported case of myoclonus [7], so they are not within the realm of normal potential side effects [3]. Here, we report a similar case in which the patient was determined, after thorough investigation, to have focal seizures linked to terbutaline sulfate syrup use.

Seizures can be caused by infectious, genetic, structural, immune, or metabolic abnormalities [8], which had to be ruled out before the seizures in this patient could be attributed to the terbutaline sulfate. Epileptic seizures were less likely to be the culprit considering the clinical picture, absence of family history, and a normal EEG in our patient, whereas an EEG in an epilepsy disorder may show epileptiform sharp waves [8]. However, we would like to state, as a limitation of the study, that due to resource unavailability, the EEG was performed after the seizures had already ceded, and we were not able to perform an MRI. A normal metabolic profile, temperature, and WBC count on the CBC ruled out the other possible causes. Iron deficiency anemia was coincidentally found, which can be a cause of febrile seizures [9], as occurred in this patient in the past, but it was ruled out to be causative in this instance when cessation of terbutaline led to cessation of the seizures prior to any iron supplementation. The one thing of concern was the low weight for age, which was <5th percentile due to the low birth weight and preterm delivery. However, other aspects of development of the child, such as social cues, were normal for her age, and thus developmental abnormalities were ruled out as the cause for the seizures [10].

Furthermore, our patient was being administered a dose of 1 ml of the 0.3 mg/ml syrup three times a day, which was around the normal single dose of 0.075 mg/kg [11], with seizures occurring only up to twice a day for the initial two days. However, when there was an increase in the terbutaline dosage to 2 ml over the past day as given by the mother, it coincided with an increased rate of seizures to several time a day over the last 24 hours and also caused a mild tachycardia. This acted in solidifying our belief that the culprit was terbutaline as it can cause seizures in overdoses [3] and the seizure increase in frequency occurred after the increase in drug dose. The syrup was immediately discontinued and the mother was warned about its side effects and that it was not recommended for use in children below the age of 12 [3]. The patient was then referred for the aforementioned testing, which came back negative for epileptiform disorders. There had been no recurrence of the seizure episodes on follow-up to discuss the test results, confirming our suspicion that terbutaline was the cause. Repeat follow-up after four months revealed no recurrence of seizures.

## Conclusions

Review of medications with new onset seizures is a standard practice, but terbutaline should be added to the list of drugs to watch out for, especially in young children. Our patient had an onset of focal seizures with terbutaline therapy with an increase in seizure frequency after a dose increase followed by a cessation of seizures on therapy cessation. With no other identifiable cause for the seizures and normal testing, it led us to conclude that terbutaline therapy lowers the threshold for focal seizures in young children. Therefore, in the future, young patients on terbutaline therapy should be monitored for seizure-like activities with a low index of suspicion, and the benefits of therapy should be weighed carefully against the consequences.
